# Fear of reinjury after acute Achilles tendon rupture is related to poorer recovery and lower physical activity postinjury

**DOI:** 10.1002/jeo2.70077

**Published:** 2024-10-31

**Authors:** Elin Larsson, Agnes LeGreves, Annelie Brorsson, Pernilla Eliasson, Christer Johansson, Michael R. Carmont, Katarina Nilsson Helander

**Affiliations:** ^1^ The Department of Orthopaedics, Sahlgrenska University Hospital Mölndal, Institute of Clinical Sciences at Sahlgrenska Academy Gothenburg University Gothenburg Sweden; ^2^ IFK Kliniken Rehab Gothenburg Sweden; ^3^ The Department of Orthopaedics, Institute of Clinical Science at Sahlgrenska Academy Gothenburg University Gothenburg Sweden; ^4^ The Department of Trauma & Orthopaedic Surgery, Princess Royal Hospital Shrewsbury & Telford Hospital NHS Trust Shropshire UK

**Keywords:** Achilles tendon rupture, Achilles tendon Total Rupture Score, fear of reinjury

## Abstract

**Purpose:**

The aim of this study was to investigate how fear of reinjury to the Achilles tendon affects return to previous levels of physical activity and self‐reported Achilles tendon Total Rupture Score (ATRS) outcomes.

**Methods:**

Data were collected from a large cohort of patients treated for an acute Achilles tendon rupture at Sahlgrenska University Hospital Mölndal between 2015 and 2020. The ATRS and additional questions concerning fear of reinjury, treatment modality, satisfaction of treatment and recovery were analyzed 1–6 years postinjury. Analysis was performed to determine the impact of fear of reinjury on patient‐reported recovery and physical activity.

**Results:**

Of a total of 856 eligible patients, 550 (64%) answered the self‐reported questionnaire and participated in the follow‐up. Of the participants, 425 (77%) were men and 125 (23%) were women. ATRS, recovery in percentage, satisfaction of treatment, recovery on a 5‐point scale and physical activity level post‐ versus preinjury were significantly related to fear of reinjury (*p* < 0.001). Of the nonsurgically treated patients, 59% reported fear of reinjury compared to 48% of the surgically treated patients (*p* = 0.024) Patients that reported fear of reinjury had a 15‐point lower median ATRS score than those who did not (*p* < 0.001).

**Conclusion:**

More than half of patients who have suffered an Achilles tendon rupture are afraid of reinjuring their tendon. Patients who reported fear of reinjury exhibited a significantly lower ATRS score. This indicates the importance of addressing psychological aspects in the treatment after this injury.

**Level of Evidence:**

Level II.

**Abbreviations**
ATR: Achilles tendon rupture
ATRS: Achilles tendon Total Rupture Score
IQR: interquartile ranges
SD: standard deviation
SU/Mölndal: Sahlgrenska University Hospital Mölndal

## INTRODUCTION

The incidence of acute Achilles tendon rupture (ATR) is increasing, with a particularly higher risk of injury among those who are middle‐aged, male and undertake a more active lifestyle [3]. Huttunen et al. presented that men are four times more likely to suffer from acute ATR than women [6]. The choice of treatment for ATR varies between hospitals, regions and countries and opinions are divided as to whether surgical or nonsurgical treatment is recommended. It is generally agreed from intervention studies that surgical repair reduces the risk of rerupture over nonsurgical treatment by approximately two to four times, although the magnitude of risk reduction may vary between studies [10, 11, 13]. However, surgical treatment can lead to a number of complications, including adhesions, iatrogenic nerve injury, pain, cosmetically unappealing scars, infection or other wound problems. It must also be considered that surgical repair does not eliminate the risk of rerupture [8]. A rupture of one's Achilles tendon can mean long sick leave with financial losses for the individual patient [16]. Furthermore, a long rehabilitation awaits and despite this, many do not regain full function [1].

Few studies have been published regarding the psychological well‐being and attitudes of patients toward recovery after an ATR and the impacts of these factors on return to physical activity [7, 14]. Jónsdóttir et al. presented that *n* = 25 patients (50%) with acute ATR, refrained from physical activity due to fear of reinjury to the Achilles tendon [7]. Patients who were afraid of new injuries had a significantly greater difference in strength between their injured leg and healthy leg compared with those who did not [7]. Olsson et al. showed that patients reporting fear had significantly worse self‐reported outcomes and physical activity 3 months after an ATR [14].

Fear of reinjury is a well‐documented concept in sports medicine as a barrier to rehabilitation. In a clinical review, Hsu et al. identified that fear of reinjury can have a negative impact on rehabilitation, recovery and subsequent successful return to sports participation [5]. The authors proposed that athletes with a high fear of reinjury would benefit most from psychologically informed practice to enhance rehabilitation. Psychologically informed practice, as described in the article, includes measuring fear of reinjury using PROM to monitor during rehabilitation [5].

The aim of this study was to investigate how fear of reinjury to the Achilles tendon affects return to previous physical activity in a large cohort. The specific questions of interest were how fear affects recovery after acute ATR and return to previous activity. The hypothesis was that a greater proportion of patients treated nonsurgically would refrain from physical activity due to fear of reinjury.

## MATERIALS AND METHODS

The study was approved by the Swedish Ethical Review Authority (dnr 2021‐01779).

This retrospective cross‐sectional study compiled the medical records for all patients who visited the emergency department with an acute ATR at Sahlgrenska University Hospital Mölndal (SU/Mölndal) between 1 January 2015 and 31 December 2020 and received the main diagnosis of S86.0 (damage to the Achilles tendon). Patients meeting these inclusion criteria were then contacted by mail 1–6 years after their initial injury.

Eight‐hundred‐and‐fifty‐six patients were invited to the study and 550 patients (64.3%) were enroled in the study. Patient demographic is presented in Table 1. The letter contained information about the study and an offer to participate. They were asked to self‐report using the Achilles tendon Total Rupture Score (ATRS) questionnaire [12], a patient‐reported outcome measure with high reliability and validity for measure outcome after treatment for an ATR. Patients were also provided additional questions regarding physical activity levels pre‐ and postinjury, treatment satisfaction, recovery (both in percent and on a Likert scale) and fear of reinjuring their Achilles tendon. When reviewing medical records, the following parameters were documented: age, sex, date of injury, date of admission to the emergency department and treatment modality.

**Table 1:** Patient demographics.

	Total (*n *= 550)	Women (*n *= 125)	Men (*n *= 425)	*p* Value
Age, years, mean (SD)	48 (14.9)	45 (14.7)	49 (14.9)	**0.006** a
BMI, mean (SD)	26 (3.8)	25.3 (4.3)	26.5 (3.6)	**0.002** a
Treatment, *n* (%)				n.s.^b^
Nonsurgery	395 (72%)	86 (69%)	309 (73%)	
Surgery	155 (28%)	39 (31%)	116 (27%)	

*Note*: Bold values are statistically significant.

Abbreviations: BMI, body mass index; *n*, number of patients; n.s., not significant; SD, standard deviation.

^a^ Student's *t* test.

^b^ Pearson's *χ* ^2^ test.

### Statistical analysis

Descriptive statistics for patient demographics and outcomes were reported as counts and proportions for categorical variables. Continuous variables were reported as means with standard deviations for normally distributed data and medians with interquartile ranges (IQR) for nonnormally distributed data. Distribution of variables was examined by visual inspection of histograms. For comparison between the two groups of patients indicating the presence or absence of fear of reinjury (yes/no), the Pearson *χ* ^2^ test was used for categorical variables. The Mann–Whitney U‐test was used to compare ATRS scores and recovery in percentage between the two groups. All tests were two‐sided, and the significance level was set at 0.05. IBM SPSS Statistics for Mac, version 28 (IBM Corp.) was used for all statistical tests.

## RESULTS

Of the 856 eligible patients, 550 (64.3%) answered the question of whether they refrained from physical activity due to fear of Achilles tendon reinjury. Of these, 56% reported a fear of reinjury to the Achilles tendon, as shown in Table 2.

**Table 2:** Fear of reinjury.

	Total (*n *= 550)	Women (*n *= 125)	Men (*n *= 425)	*p* Value
Fear, *n* (%)				n.s.^a^
Yes	308 (56%)	71 (57%)	237 (56%)	
No	242 (44%)	54 (43%)	188 (44%)	

Abbreviation: *n*, number of patients.

^a^ Pearson's *χ* ^2^ test.

**Figure 1:**
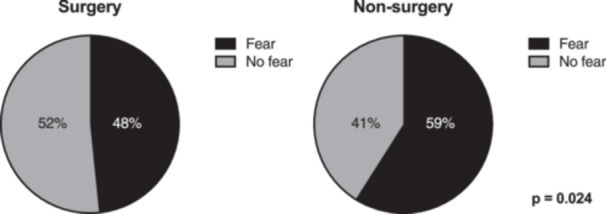
Distribution of patients treated with surgery (left) and nonsurgical treatments (right) reporting fear and no fear of Achilles tendon reinjury. Pearson's *χ* ^2^ test.

**Figure 2:**
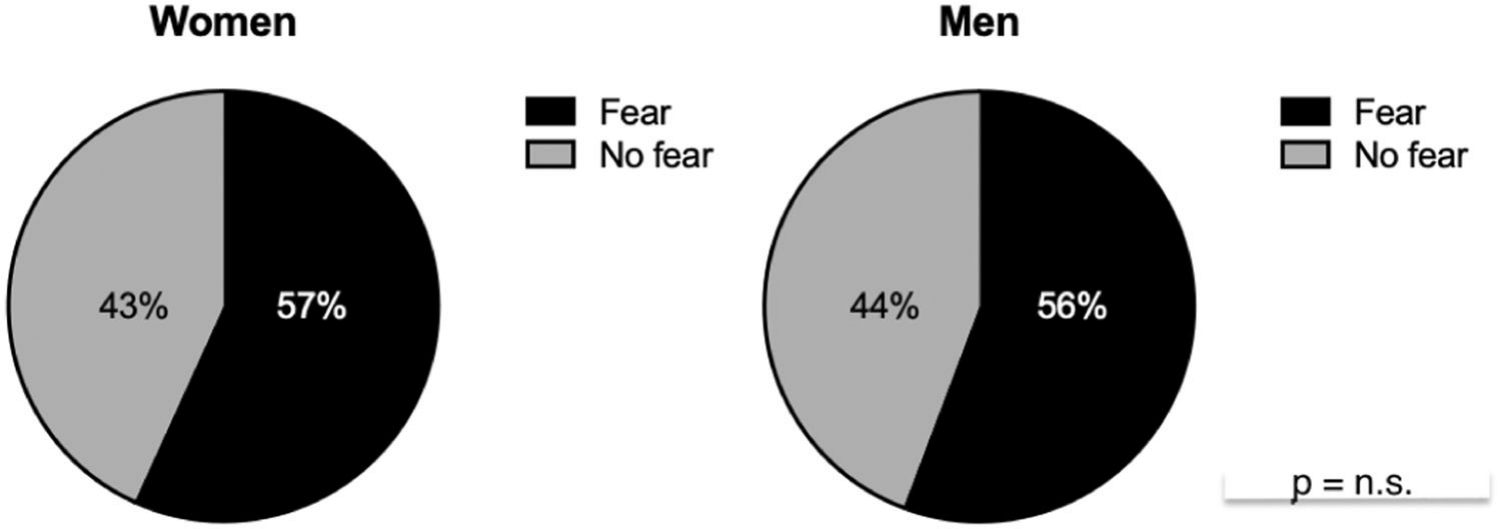
Distribution of women (left) and men (right) reporting fear and no fear of Achilles tendon reinjury. Pearson's *χ* ^2^ test.

Treatment, ATRS, satisfaction, recovery and current activity compared to before were significantly related to fear (Figure 1, Table 3). There was no significant difference between women and men reporting fear of reinjury as seen in Figure 2. The group of patients that reported fear of reinjury had a 15‐point lower ATRS score compared to the group that did not report fear of reinjury. Recovery expressed, as a percentage from 0% to 100%, was significantly lower among patients experiencing fear of reinjury (Table 3).

**Table 3:** Treatment, recovery, activity and satisfaction outcomes.

	Fear (*n *= 308)^a^	No fear (*n *= 242)^b^	*p* Value
ATRS score, median (IQR)	76 (55–90)	91 (78–97)	**<0.001** a
Recovery, median % (IQR)	80 (70–90)	90 (82–98)	**<0.001** a
Treatment satisfaction, *n* (%)			**<0.001** b
Completely satisfied	109 (35.4%)	146 (60.3%)	
Somewhat satisfied	112 (36.4%)	69 (28.5%)	
Neither satisfied nor dissatisfied	52 (16.9%)	19 (7.9%)	
Somewhat dissatisfied	22 (7.1%)	4 (1.7%)	
Dissatisfied	13 (4.2%)	4 (1.7%)	
Recovery, *n* (%)			**<0.001** b
To full extent	48 (16%)	106 (44%)	
To a large extent	183 (59%)	110 (45%)	
Neither	27 (8.8%)	11 (4.5%)	
To a small extent	48 (16%)	15 (6.2%)	
Not at all	2 (0.6%)	0 (0%)	
Current activity compared to before			**<0.001** b
Much more active	11 (3.6%)	8 (3.3%)	
Somewhat more	21 (6.8%)	26 (11%)	
Same	83 (27%)	126 (52%)	
Somewhat less	142 (46%)	64 (26%)	
Much less active	51 (17%)	18 (7.4%)	

*Note*: Bold values are statistically significant.

Abbreviations: ATRS, Achilles tendon Total Rupture Score; IQR, interquartile range.

^a^ Mann–Whitney U test.

^b^ Pearson's *χ* ^2^ test.

**Figure 3:**
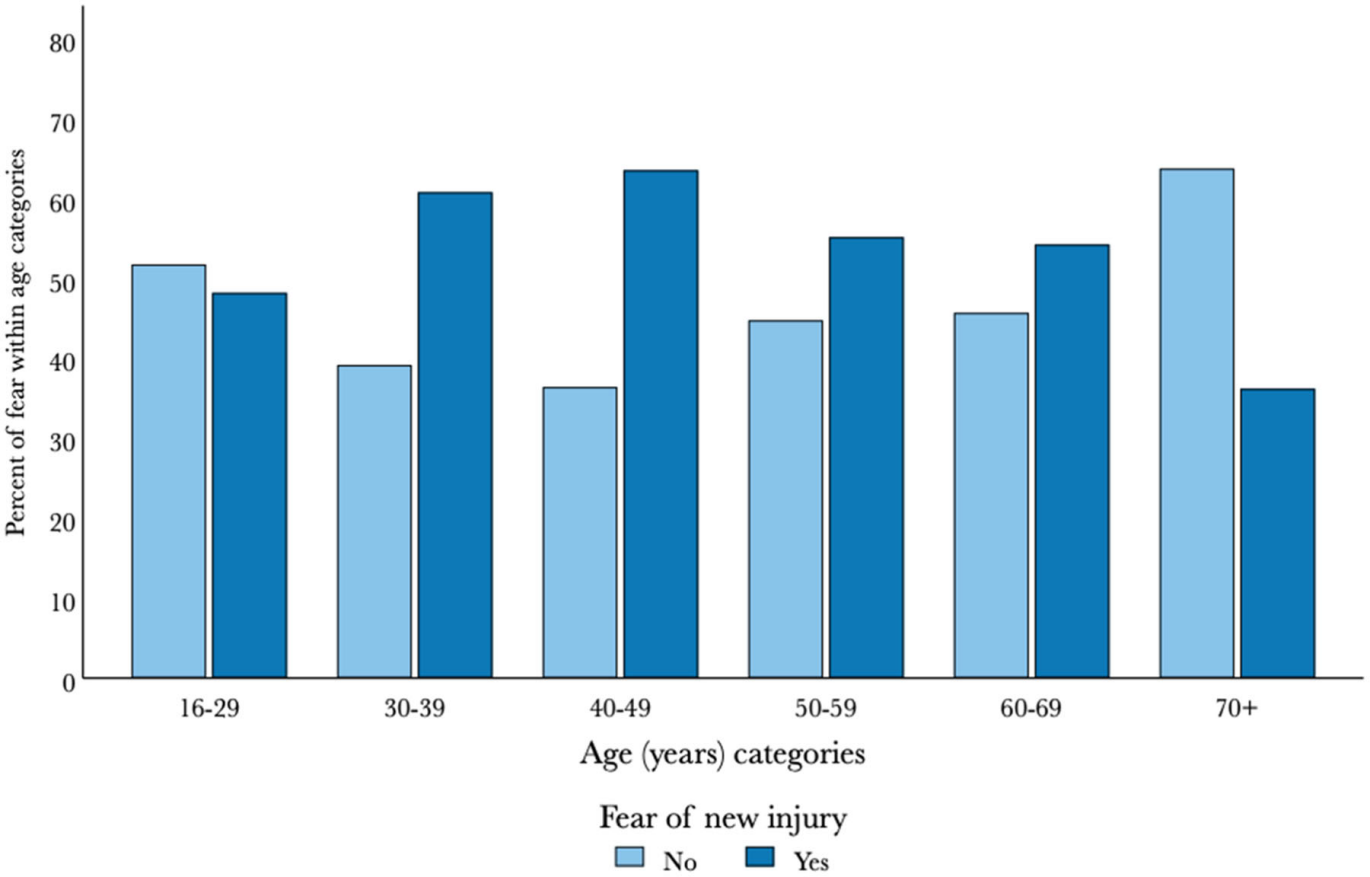
Proportion of patients reporting fear of Achilles tendon reinjury within each age category. The percentages reporting yes/no sum up to 100% in each age category. A *χ* ^2^ test for association indicates an association between fear and age categories (*p* = 0.008).

There was a significant association between reported fear of reinjury and the age categories (*p *= 0.008). The largest proportion reporting fear of reinjuring the Achilles tendon was found in the 30–39 and 40–49 age categories. The lowest proportion who reported fear of reinjuring the Achilles tendon was among patients aged 70 years or older (Figure 3).

## DISCUSSION

The most important finding of this cross‐sectional cohort study is that 56% of the 550 patients who suffered from an acute ATR reported a fear of reinjuring their Achilles tendon. Most patients who experienced fear of reinjury did not return to the same level of physical activity as before their injury, were less satisfied with treatment, and had worse recovery and ATRS outcomes compared to those reporting no fear of reinjury. Furthermore, a larger proportion of nonsurgically treated patients reported fear of reinjury. The results of the present study are in line with previous studies that investigated the frequency of fear of reinjury among patients suffering from a musculoskeletal injury [7, 9]. However, the present study is based on a larger cohort than previously published reports. Jónsdóttir et al. reported that 50% out of 25 included patients with an ATR felt such a high level of fear of rerupture that they refrained from physical activity [7]. Similarly, Kvist et al. found that 53% of the 62 who had sustained an injury to the ACL of the knee returned to their previous activity levels. Of those who did not return, the primary reason was fear of reinjury [9].

The findings of the present study indicate that the majority of the patients expressed satisfaction with the treatment of their injured Achilles tendon. However, it was observed that patients who resumed participation in sports activities without reporting a fear of reinjury demonstrated a higher level of satisfaction than those who did report such a fear. Moreover, there was a significant correlation between fear of reinjury and recovery. There was a large, significant difference of 15 points in ATRS scores between patients who reported fear of reinjury and those who did not. Similarly, patients who felt fear of reinjury had 10% lower median recovery scores than those who did not. Consistent with ATRS findings, only 27% of patients with fear of reinjury reported a return to the same preinjury activity level compared with 52% of those without fear (*p* < 0.001). The reasons for these large differences require further investigation. In future studies, it is of interest to gain patient perspectives regarding fear to better understand impact on daily life due to insufficient recovery.

The present study showed significant discrepancy in the prevalence of fear of reinjury between patients who underwent nonsurgical and surgical treatment (59% vs. 48%; *p* = 0.024). Previously, Grevnerts et al. found that the inability to perform physical activity and fear of increased symptoms are strong factors in the patient's choice of surgical treatment over nonsurgical treatment for ACL rupture [4]. These factors could be influenced by a patient's belief that surgery entails a lower risk of future problems with pain and a lower risk of reinjury. Filbay et al. found that patients who proceeded to surgical treatment of ACL ruptures reported greater fear of reinjury before treatment compared with nonsurgically treated patients [2]. The results of our study, in alignment with those of Grevnerts et al. [4] and Filbay et al. [2], indicate that fear plays a significant role in both treatment selection and the return to baseline function and daily activities. With surgery, patients may feel less afraid of rerupturing their Achilles tendon due to the fact that the tendon ends are surgically repaired together, and the strength of the tendon is thus stronger. This psychological effect makes surgical treatment a predictor of less fear. Nevertheless, there is no strong evidence that suggests surgical treatment provides a better functional outcome [10].

In our cohort, the largest proportion of patients who reported a fear of reinjury was observed in the 30–39 and 40–49 age groups, while the 70+ age group exhibited the smallest proportion of patients fearful of reinjury. This trend could be because the elderly usually have lower demands and lower levels of physical activity, and, therefore, fewer opportunities to abstain from physical activity. In addition, the elderly may be more concerned about other more serious diseases than reinjury to their Achilles tendon. Another explanation could be that most patients aged 30–49 years are employed and/or have children and, therefore, feel that they cannot afford to lose time due to reinjury, hence they might experience greater fear.

There is a need to determine why such a large percentage of patients feel afraid of injuring themselves again and, therefore, avoid physical activity. And several questions remain as to why and when fear of reinjury arises after an acute ATR. There is also a need to characterize the degree to which patients are affected in their everyday life because of their fear and whether they have stopped doing certain activities compared to preinjury simply because of fear. In addition to patient‐related factors (e.g., personality traits), fear of reinjury may, unfortunately, be supported or even amplified by comments and mannerisms of treating clinicians during the recovery process. Collectively, the results of this study emphasize the importance of treating clinicians in encouraging patients to not be afraid of pursuing physical activity or sports. In their investigation of psychological factors during the rehabilitation of ATR, Slagers et al. [15] showed that fear of movement decreased and readiness to return to sport improved over time. This is in line with our interpretation that psychological factors could affect rehabilitation, where physiotherapists have an essential role in terms of screening for and addressing fear. Going forward, rehabilitation that includes psychological aspects could be a valuable addition to physical rehabilitation to overcome fear and encourage physical activity after injury, thus, preventing patients from getting stuck in a vicious cycle of fear and lack of physical activity. In the orthopaedic community, individualized ‘treatment’ is commonly seen as an approach to optimize outcomes after an injury. Fear of reinjury could be an important component of an individualized choice of treatment during the rehabilitation process for acute ATR.

The large cohort size is one of the strengths of this study. Previous studies in the same field have been carried out on much smaller cohorts. Another strength was the use of the ATRS, an injury‐specific, validated and reliability‐tested patient‐reported outcomes measure, to evaluate recovery in patients treated for a total ATR. Furthermore, all patients included in the present study were managed at one centre, which means that the same local guidelines were followed regarding the assessment of injury, ATRS and treatment choice reducing the heterogeneity of the cohort. But this can also be regarded as a limitation since the results could be less generalizable and applicable to other settings and in other countries with different working methods and guidelines. Another limitation is the retrospective nature of this study and the effect of recall bias. The questionnaire was sent to patients so that at least 1 year had passed since injury, and the period between the injury and questionnaire completion varied from 1 to 6 years. Recall bias can affect the patients' answers as it can be difficult to remember the time before injury. Nevertheless, previous studies identified no significant differences in ATRS scores 2 years after injury or several years after injury [1]. Related to the long study period, only 66% of patients who were invited accepted and participated in the follow‐up study and answered the questionnaires. The moderate response rate could pose a risk of selection bias in that patients who felt completely satisfied with their treatment and recovery chose to answer the questionnaire to a greater extent than those who did not feel satisfied. Finally, the study questionnaire included only one question related to fear of reinjury. This question was a yes/no question, and there was no possibility for patient reporting the level of fear. Future studies could ask more questions regarding fear and the reasons for perceived fear. Studies where patients are interviewed could help explore the topic further.

The findings of this study highlight the crucial importance of inquiring about the potential fear of reinjury during the course of both medical consultations—with the orthopaedic surgeon and the physiotherapist—and of addressing this issue in a comprehensive and sensitive manner.

## CONCLUSIONS

More than half of patients affected by acute ATR are afraid of reinjuring their Achilles tendon. Patients who experienced fear of reinjury have a significantly worse self‐estimated recovery as measured by the ATRS. The findings of this study emphasize the importance of taking patients' fear of injury seriously.

## AUTHOR CONTRIBUTIONS

Elin Larsson, Agnes LeGreves, Annelie Brorsson, Pernilla Eliasson, Christer Johansson, Michael R. Carmont and Katarina Nilsson Helander participated in the design of the study. Elin Larsson, Katarina Nilsson Helander and Christer Johansson performed the data processing and statistical analysis. All of the authors have contributed to the manuscript.

## CONFLICT OF INTEREST STATEMENT

The authors declare no conflict of interest.

## ETHICS STATEMENT

Approval was provided by the Swedish Ethical Review Authority, dnr 2021‐01779. All patients that were included in the study provided written consent for enrolment.

## Data Availability

The data sets generated and/or analyzed during the current study are not publicly available due to confidential information but are available from the corresponding author on reasonable request.
